# Supplementary figures and images for: Role of ADME genes in breast cancer prognosis: an analysis of risk scoring models based on multi-omics data
Source: Front Oncol. 2025 Jun 4;15:1582862. doi: 10.3389/fonc.2025.1582862 (PMC12174373; doi:10.3389/fonc.2025.1582862)

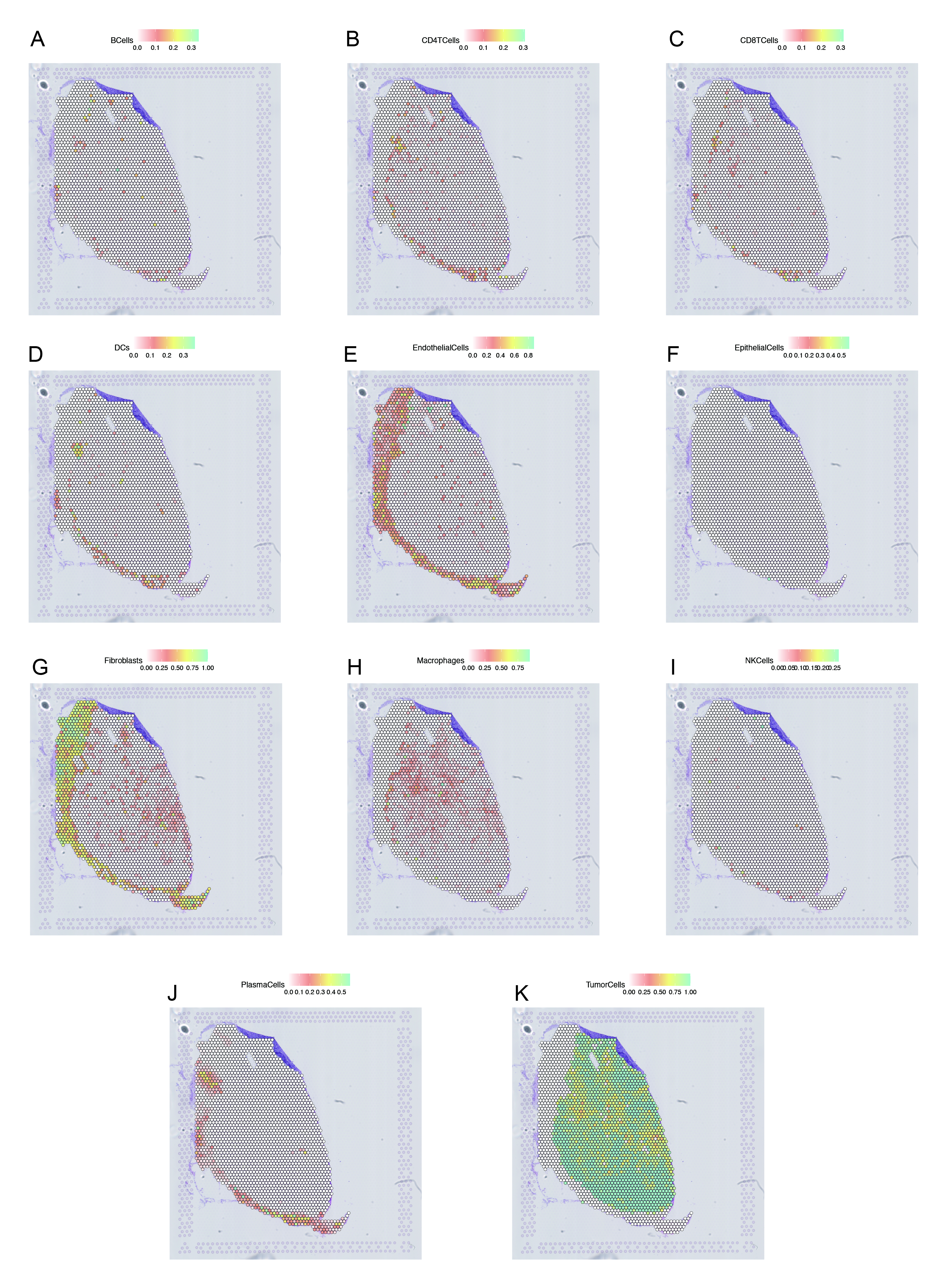

Supplement: Supplementary Figure 1 — 9 distinct cell types in space of BC. [file Image1.tif]
